# Focal lesion size poorly correlates with motor function after experimental traumatic brain injury in mice

**DOI:** 10.1371/journal.pone.0265448

**Published:** 2022-03-16

**Authors:** Johannes Walter, Jannis Mende, Samuel Hutagalung, Martin Grutza, Alexander Younsi, Guoli Zheng, Andreas W. Unterberg, Klaus Zweckberger

**Affiliations:** Department of Neurosurgery, Heidelberg University Hospital, University of Heidelberg, Heidelberg, Germany; Uniformed Services University, UNITED STATES

## Abstract

**Background:**

It remains unclear whether neurobehavioral testing adds significant information to histologic assessment of experimental traumatic brain injury (TBI) and if automated gait assessment using the CatWalk XT^®^, while shown to be effective in in the acute phase, is also effective in the chronic phase after experimental TBI. Therefore, we evaluated the correlation of CatWalk XT^®^ parameters with histologic lesion volume and analyzed their temporal and spatial patterns over four weeks after trauma induction.

**Methods:**

C57Bl/6 mice were subjected to controlled cortical impact (CCI). CatWalk XT^®^ analysis was performed one day prior to surgery and together with the histological evaluation of lesion volume on postoperative days one, three, seven, 14 and 28. Temporal and spatial profiles of gait impairment were analyzed and a total of 100 CatWalk XT^®^ parameters were correlated to lesion size.

**Results:**

While in the first week after CCI, there was significant impairment of nearly all CatWalk XT^®^ parameters, impairment of paw prints, intensities and dynamic movement parameters resolved thereafter; however, impairment of dynamic single paw parameters persisted up to four weeks. Correlation of the CatWalk XT^®^ parameters with lesion volume was poor at all timepoints.

**Conclusion:**

As CatWalk XT^®^ parameters do not correlate with focal lesion size after CCI, gait assessment using the CatWalk XT^®^ might add valuable information to solitary histologic evaluation of the injury site. While all CatWalk XT^®^ parameters can be used for gait assessments in the first week after CCI, dynamic single paw parameters might be more relevant in the chronic phase after experimental TBI.

## Introduction

With more than 80.000 traumatic brain injury (TBI) related deaths per year in Europe alone and up to one third of the TBI patients not achieving a full recovery at six months after the injury, the disease remains a major medical as well as socioeconomic challenge in developing as well as developed countries [1–3]. Despite its significant contribution to morbidity and mortality, treatment options, especially those directly interfering with the specific pathophysiology of TBI, are still very limited and remain strictly symptomatic or experimental [4, 5].

To overcome this dilemma, many innovative treatment approaches have been evaluated in a multitude of different preclinical models of TBI and promising results have been described; however, none of these treatments has shown a significant benefit in a large randomized controlled clinical trial so far [6–8]. One of the main issues contributing to the failure of translating promising preclinical treatment approaches to the clinical setting is insufficient preclinical outcome assessment leading to potential overestimation of treatment effects: Primary clinical trial endpoints have recently been shifted from solely assessing radiological or monitoring parameters to evaluating functional outcome parameters such as the Extended Glasgow Outcome Scale as these parameters have been recognized as being more predictive for the patient`s quality of life. However, preclinical TBI studies mainly focus on histopathological parameters such as contusion volume as primary outcome measures. A reason for this mismatch in outcome measures might be the notorious difficulty of objectively and rater independently evaluating gait and motor function in preclinical TBI models in rodents.

The controlled cortical impact model is one of the most frequently used models of experimental TBI [9–24]. Even though a plethora of neurobehavioral tests is available for assessment of gait and motor function after CCI, data on the correlation of such neurobehavioral tests and histopathologic injury parameters are scarce. Therefore; it remains unclear if neurobehavioral testing adds significant extra information to outcome assessed by histopathological parameters and if it significantly contributes to assessment of treatment effects overall. Given the possibility that, for instance, impaired motor function is merely the direct result of focal histological damage, histological outcome assessment might be sufficient and extensive neurobehavioral testing thus time- and cost-inefficient.

The CatWalkXT^®^ has been devloped to automatically and observer independently assess gait and motor function in rodents. It has been used in a variety of preclinical models of traumatic and non-traumatic neurologic conditions such as Parkinson’s disease, stroke, peripheral nerve injury, spinal cord injury as well as traumatic brain injury [25–31]. While the CatWalkXT^®^’s value in gait assessment in experimental models of spinal cord injury and peripheral nerve injury has been well documented, it’s value in the context of gait assessment in preclinical TBI in rodents remained unclear. However, in a previous study, we recently validated the CatWalkXT^®^ as an excellent rater independent automated test of gait and motor function in the acute phase after CCI in mice, identifying it as an excellent tool for testing these domains of neurobehavioral function specifically in the CCI model in rodents [16]. Yet, it remains unclear if there is a robust correlation between structural damage and gait and motor function after experimental TBI in mice and thus, if the effects of a treatment can be thoroughly evaluated focusing on histological outcome parameters or if more focus should be directed towards neurobehavioral testing in preclinical TBI studies.

Therefore, in the current study, we evaluated the correlation between histopathologic contusion volume and gait and motor function analyzed with the CatWalk XT^®^ within the first four weeks after CCI induction in order to assess its potential use in the chronic phase after CCI as well as its additional value compared to sole histological injury evaluation.

## Materials and methods

### Animals

A total of 45 male C57Bl/6 mice (Charles River Laboratories, Sulzfeld, Germany) aged six to eight weeks with a body weight of 20-25g were used for the experiments and randnomly distributed into five experimental groups (one-, three-, seven-, 14- and 28-day group; n = 9 per group). Operative mortality was 0% as no mouse died in direct relation to the surgical procedure; however, two mice from the one- and three-day group were excluded from data analysis as they had to be euthanized prematurely after meeting termination criteria (the animals had lost more than 30% of the initial body weight within the first 24 hours after trauma induction) leaving seven mice available for lesion analysis in the one- and three-day group. One mouse in the 28-day group had to be excluded from CatWalk analysis as no runs eligible for analysis could be obtained at the resprective timepoints.

Animals were kept at a 12h-day/12h-night cycle and had access to food and water ad libitum. All procedures were reviewed and approved by the Animal Care Committee of the federal government (Regierungspräsidium Karlsruhe, approval number G-296/19). Postoperative health screens and hygiene management checks were performed in accordance with Federation of European Laboratory Animal Science Associations guidelines and recommendations [32].

### Controlled Cortical Impact (CCI) procedure

The CCI procedure was carried out as described before [9, 17, 33]. Mice were analgized by applying a single subcutaneous bolus injection of buprenorphine (Temgesic^®^, Indivior Europe Ltd, Dublin, UK) at a dose of 0.1mg/kg 30 minutes prior to surgery as well as subcutaneous injections of Carprofen (Norbrook Laboratories, Newry, Northern Ireland) at a dose of 5mg/kg 12 and 24 hours after trauma induction. Subcutaneous injections were placed on the back of the animals hence not interfering with the free movement of the animals. After analgesia was established, anesthesia induction was performed applying a mix of Isoflurane/oxygen/air (5%/30%/65%) in an induction chamber for 45 seconds. As soon as deep anesthesia was established, animals were placed into a stereotactic head frame (Kopf Instruments, Tujunga, California, USA) and anesthesia was continued using 1%-1.5% Isoflurane/30% oxygen/69% air. Animals were placed on a feedback-controlled heating pad adjusted to 37°C for the time of surgery in order to prevent potential neuroprotective hypothermia. After a midline skin incision, a right parietal craniotomy extending form the superior sagittal suture to the superior temporal line laterally and form the lambdoid suture to bregma anteror-posteriorly was performed using a high-speed drill under permanent cooling with saline and preserving the integrity of the dura mater. After elevation of the bone flap, the pneumatic driven impactor tip (2mm diameter) was placed perpendicular to the surface of the cortex. CCI was then induced using the following parameters: impact depth 1mm, speed 8m/s, dwell time 150ms. The mentioned parameters were chosen as they have been used in previous studies and result in highly reproducible moderate TBI [9–19]. After CCI induction, cranioplasty was performed immediately by repositioning the autologous bone flap and sealing the craniotomy with histoacrylic glue (Histoacryl^®^, B. Braun Melsungen AG, Melsungen, Germany). Finally, skin closure was performed with 4–0 Vicryl interrupted sutures. Postoperatively, the animals`cages were placed on a heating plate at 34°C for one hour to prevent potentially neuroprotective hypothermia.

### CatWalkXT^®^

In the current study the CatWalk XT^®^ Version 10.6 (Noldus Information Technology, Wageningen, The Netherlands) was used for gait assessments. Before performing CatWalk XT^®^ testings, all animals were habituated to the new environment for one week. Animals were first exposed to the CatWalk XT^®^ at baseline data collection which was one day prior to trauma induction. The CatWalk XT^®^ was then performed on day one, three, seven, 14 and 28 after CCI, respectively, depending on the experimental group. To improve animal compliance and create a set up as similar to the animals`natural environment as possible, the tests were conducted in a room specially dedicated to CatWalk XT^®^ testing with dimmed light and at reduced noise. The CatWalk XT^®^ analysis was repeated three times at each time point to account for intraindividual variability and means for each animal were calculated.

**Fig 1:**
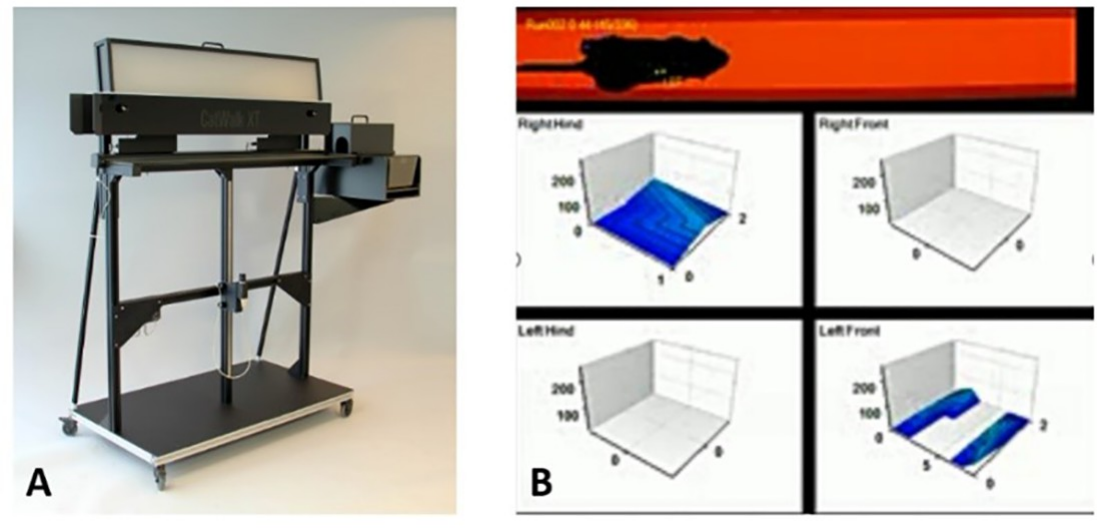
Overview of the CatWalkXT^®^ automated gait analysis system. The hood is elevated to obtain a better overall impression of the walkway. For gait analysis, the hood is closed creating a tunnel guiding the animals direction of movement (A, image provided Noldus Information Technology, Wageningen, The Netherlands). Fruthermore, an example of automated CatWalkXT^®^ footprint recognition is provided (B).

The CatWalk XT^®^ itself consists of a horizontal glass plate of 1.3m in length covered by a removable tunnel creating a dimmed light on the walkway. After being positioned at the beginning of the walkway, mice traverse the plate towards the home cage voluntarily. A green LED light is emitted inside the glass plate and internally reflected, sparing those areas where contact with the glass plate is made. If the paws touch the glass, light is refracted on the opposite side. A high-speed color camera mounted directly underneath the glass plate automatically detects these illuminated areas and automatically sends the detected data to a computer where the data is automatically analyzed by the CatWalk XT^®^ software (Fig 1).

In the current study, after habituation to the new surroundings, animals completed a minimum of three nonstop runs, qualified for CatWalk XT^®^ analysis. A maximum speed variation of 60%, a camera gain of 16.99 dB and a detection threshold of 0.1 a.U. were used as calibration parameters. No set time limit to complete a single run was used. The same calibration parameters were used for every mouse. In most cases conducting only three runs in total was sufficient in order to obtain three runs eligible for analysis; however, the maximum number of runs that had to be conducted was five. We did not observe any learning effect between the runs that could have impaired the comparison between animals, that had to complete five runs, and those, that only had to complete three runs.

Automated footprint recognition was reviewed manually to improve data quality. The CatWalk XT^®^ software separately analyzed a combination of 23 dynamic and static parameters for each paw. In addition, *Print Positions* of the left and right paws as well as *Base of Support* of front and hind paws were analyzed. Finally, *Run Speed*, *Run Duration*, *Run Variation* and *Body Speed Variation* were determined. Therefore, a total of 100 parameters were automatically recorded and analyzed by the CatWalk XT^®^ software. Table 1 gives an overview of the parameters determined by the CatWalk XT^®^ analysis.

**Table 1:** Parameters automatically analyzed by the CatWalk XT^®^ software.

Parameter	Description
***Static Parameters***	
Min Intensity	Min Intensity is the minimum Intensity of the complete paw.
Max Intensity	Max Intensity is the maximum Intensity of the complete paw.
Mean Intensity	Mean Intensity is the Mean Intensity of the complete paw.
Mean Intensity of the 15 most intense pixels	Mean Intensity of the 15 pixels of a paw with the highest intensity.
Max Intensity at	Max Intensity at (s) is the time in seconds since the start of the run that the maximum intensity is measured. Max Intensity at (%) is Max Intensity at (s) relative to Stand.
Max Contact Max Intensity	Maximum Intensity at Max Contact of a paw.
Max Contact Mean Intensity	Mean Intensity of a paw at Max Contact.
Max Contact Area	Max Contact Area is the maximum area of a paw that comes into contact with the glass plate.
Max Contact Area at	Max Contact At (s) is the time in seconds since the start of the run that a paw makes maximum contact with the glass plate. Max Contact At (%) is Max Contact At (s) relative to Stand of a paw.
Print Area	Print Area is the surface area of the complete print.
Print Width	Print Width is the width (vertical direction) of the complete paw print.
Print Length	Print Length is the length (horizontal direction) of the complete print.
***Dynamic Parameters***	
Swing Speed	Swing Speed is the speed of the paw during a Swing.
Body Speed	The Body Speed is calculated by dividing the distance that the animal’s body traveled from one initial contact of that paw to the next by the time to travel that distance.
Swing	Swing (s) is the duration in seconds of no contact of a paw with the glass plate.
Step Cycle	Step Cycle is the time in seconds between two consecutive Initial Contacts of the same paw.
Stand	Stand (s) is the duration in seconds of contact of a paw with the glass plate.
Duty Cycle	Duty Cycle (%) expresses Stand as a percentage of Step Cycle.
Stand Index	Stand Index is a measure for the speed at which the paw loses contact with the glass plate.
Stride Length	Stride Length is the distance between successive placements of the same paw.
Single Stance	Single Stance is the duration of ground contact for a single hind paw.
Initial Dual Stance	Dual Stance is the duration of ground contact for both hind paws simultaneously.
Terminal Dual Stance	Terminal Dual Stance is the second step in a Step Cycle of a hind paw that the contralateral hind paw also makes contact with the glass plate.
Average Run Speed	The average speed of the recorded run.
Run Duration	The duration of the recorded run.
Run maximum variation	The maximum variation in walking speed in the recorded run.
Body Speed Variation	Body Speed Variation (%) is calculated by dividing the absolute difference between the Body Speed and the Average Speed of a run by the Average Speed.
***Combined Paw Parameters***	
Base of Support (BOS) front paws	Base of Support (BOS) front paws is the average width between the front paws.
Base of Support (BOS) hind paws	Base of Support (BOS) hind paws is the average width between the hind paws.
Print Positions left paws	Print Positions left paws is the distance between the position of the hind paw and the position of the previously placed front paw on the left side in the same Step Cycle.
Print Positions right paws	Print Positions right paws is the distance between the position of the hind paw and the position of the previously placed front paw on the right side in the same Step Cycle.

### Nissl staining

**Fig 2:**
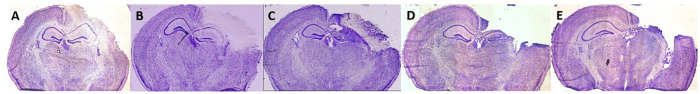
Examples of coronal sections on days one (A), three (B), seven (C), 14 (D) and 28 (E).

After sacrificing the animals by cervical dislocation at the respective timepoints one, three, seven, 14 and 28 days after CCI, brains were removed and shock frozen on dry ice. Thereafter, 16 frozen sections of 10μm in thickness were prepared every 300μm starting behind the olfactory bulb using a cryostat (Leica CM3050S, Leica Biosystems, Germany). The sections were then stained according to the Nissl protocol and photographed at 12.5-fold magnification (LSM 700; *Carl-Zeiss*, *Germany*, Fig 2). Thereafter, the areas of the contralateral hemisphere as well as the preserved tissue of the contused hemisphere were determined for every section using the ImageJ image analysis software (National Institute of Health, Bethesda, USA). Finally, the contused area of every section was calculated subtracting the preserved tissue of the contused hemisphere from the total area of the contralateral hemisphere, thereby accounting for pericontusional atrophy over the course of the experiment. Contusion volume was then determined using the following formula:

$${\text{V}}_{\text{n}}={\text{A}}_{1}\;\text{x}\;0,3+{\text{A}}_{2}\;\text{x}\;0,3\ldots +{\text{A}}_{14\;}\text{x}\;0,3$$

### Data analysis

All results are presented as means ± standard error of mean (SEM). For statistical comparison of CatWalk XT^®^ parameters to preoperative status a paired t-test was performed, if Shapiro-Wilk test for normality was passed. For comparison of non-parameteric CatWalk XT^®^ parameters, a Wilcoxon-Signed-Rank test was performed. Means of lesion volume between multiple groups were analyzed using one-way ANOVA followed by post-hoc Tukey-HSD-tests. Linear regression analysis was performed to assess the correlation of the lesion volume with the 100 different CatWalk XT^®^ parameters; results of these analyses are presented as R^2^-values to show the goodness of fit. For correlation analysis between two continuous variables, Pearson correlation coefficient was determined. Statistical significance was defined as p<0.05. All statistical analyses were performed after consultation with the Institute of Medical Biometry of the University of Heidelberg using SigmaStat data analysis software (SigmaPlot 12.0, Jandel Scientific, Erkrath, Germany).

## Results

### Lesion volume

**Fig 3:**
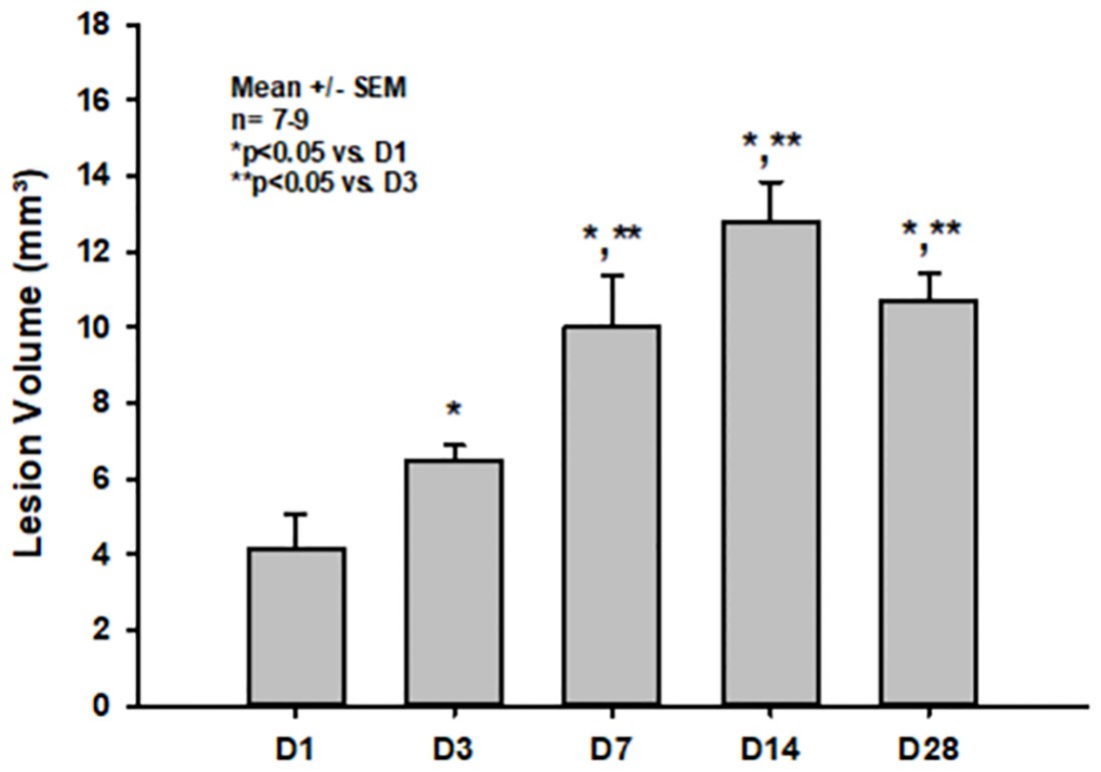
Lesion volume on days one thru 28 after CCI.

The lesion volume significantly increased within the first two weeks after CCI (3.24 +/- 2.78 mm^3^, 6.47 +/- 1.10 mm^3^ and 10.03 +/- 4.00 mm^3^ at days 1,3 and 7, respectively; p = 0.01 and p<0.001 vs. day 1 for days 3 and seven, respectively) reaching the maximum of 12.80 +/- 3.14 mm^3^ at day 14 (p<0.001 vs. d1). Thereafter, the lesion volume slightly but non significantly decreased to 10.71 +/- 2.09 mm^3^ on day 28 (Fig 3). Lesions mainly involved the right parietal cortex and subcortical areas, however, with increasing lesion volumes over time, involvement of the corpus callosum could as well as the hippocampus also be noticed (Fig 2).

### CatWalk XT^®^ parameters

#### Paw prints

**Fig 4:**
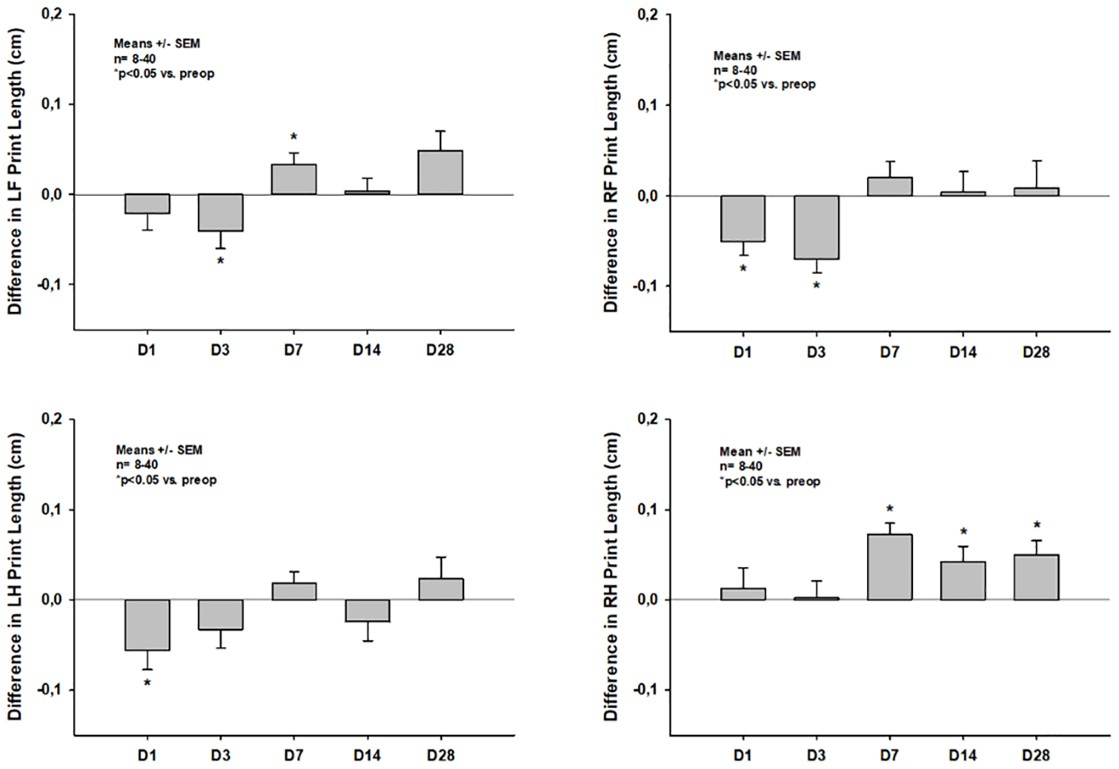
Differences in *print length* of the four single paws compared to pretrauma status on days one thru 28 after CCI.

Impairment of single paw print parameters differed between the respective diagonal opposite paw pairs: In the left hindpaw and right frontpaw print parameters significantly decreased within the first three days after CCI and returned to pretrauma levels thereafter (e.g. *print length* -0.06 +/- 0.01 cm, p = 0.012 vs. pretrauma for the left hindpaw on day one and -0.03 +/- 0.01 cm, p<0.001 vs. pretrauma for the right frontpaw on day three, respectively), whereas left frontpaw and right hindpaw print parameters were altered significantly on days one and three after trauma, but were significantly increased compared to pretrauma values on days seven, 14 and 28 (e.g. *print length* 0.07 +/- 0.06 cm, 0.04 +/- 0.07 cm and 0.05 +/-0.04 cm; p<0.001, p = 0.023 and p = 0.015 vs. pretrauma for the right hindpaw on days seven, 14 and 28, respectively). *Print length* of all four paws is shown as an example for a single paw print parameter in Fig 4.

#### Paw intensities

**Fig 5:**
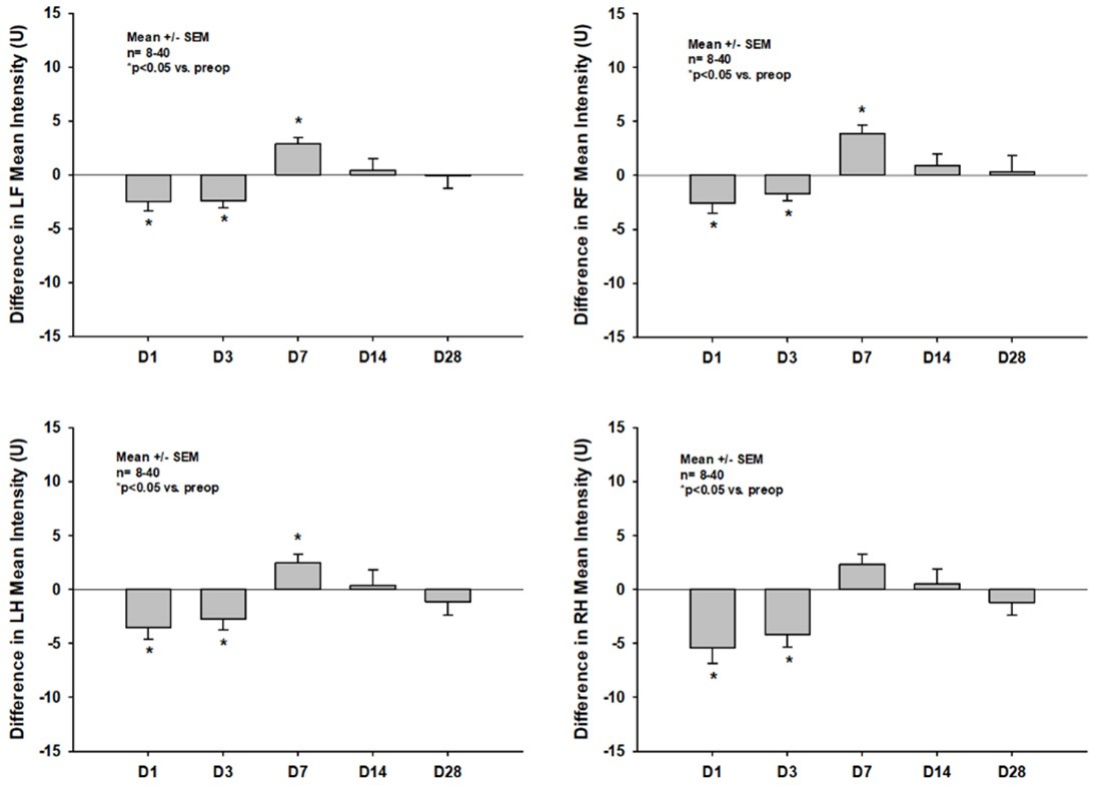
Differences in *mean intensity* of the four single paws compared to pretrauma status on days one thru 28 after CCI.

A similar pattern of impairment of the paw intensities could be seen throughout all CatWalk XT^®^ single paw intensity parameters; as an example, differences of the *mean paw intensities* compared to the pretrauma status are shown in Fig 5: While the *mean paw intensity* significantly decreased within the first three days after CCI (e.g. -3.51 +/- 6.71 U and -2.74 U +/- 5.67 U, p = 0.003 and p = 0.009 vs. pretrauma for the left left hindpaw on days one and three, respectively), it significantly increased on day seven (e.g. 2.50 +/- 3.99, p = 0.004 vs pretrauma for the left hindpaw) before returning to the pretrauma status on days 14 and 28. Impairments were more pronounced in the hindpaws as would be expected in a model of high parietal trauma location.

#### Dynamic single paw parameters

**Fig 6:**
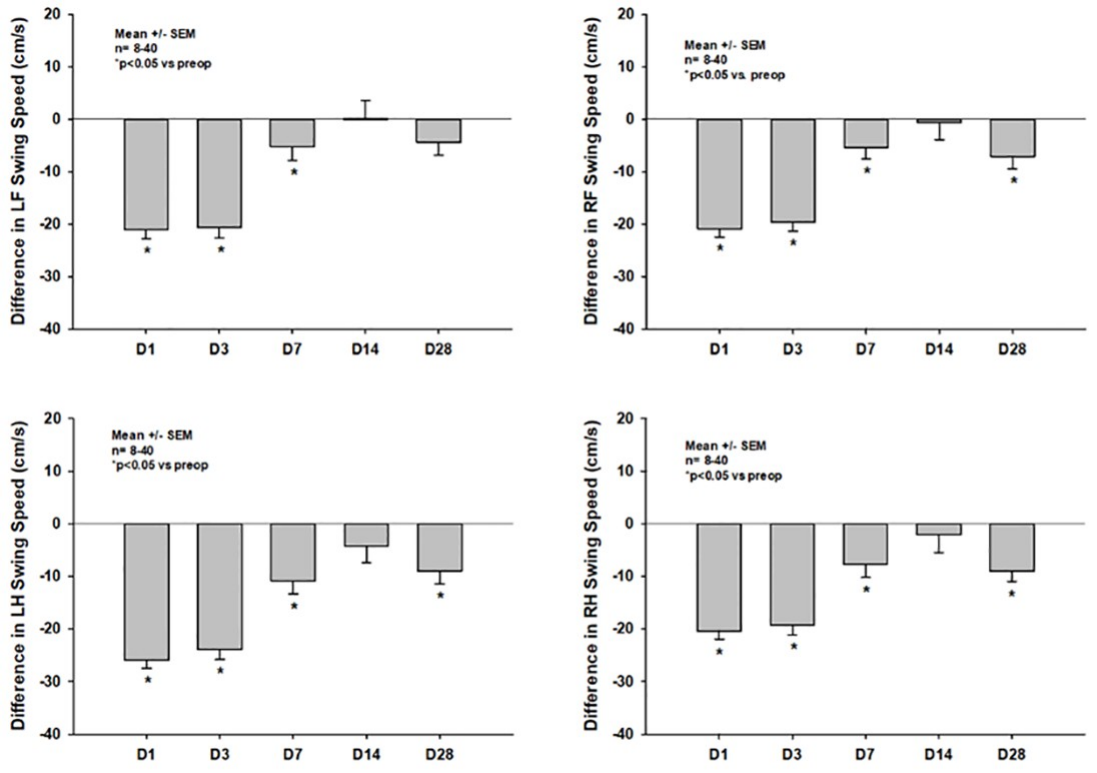
Differences in *swing speed* of the four single paws compared to pretrauma status on days one thru 28 after CCI.

In contrast to paw intensities and paw prints, dynamic single paw parameters showed significant prolonged impairment throughout the first four weeks after CCI; as an example, data on the *swing speed* is shown in Fig 6. The most significant impairment of *swing speed* was seen in the left hindpaw, as would be expected due to the right parietal trauma location, and within the first three days after trauma induction (-25.97 +/- 9.36 cm/s and -23.90 +/- 10.79 cm/s, p<0.001 vs. pretrauma each for the left hindpaw on days one and three). Afterwards, animals significantly recovered; however, they still showed significant impairments compared to preoperative status (e.g. -10.85 +/- 12.29 cm/s and -8.97 +/- 7.12 cm/s; p<0.001, and p = 0.008 for left hindpaw on days seven and 28, respectively).

#### Dynamic movement parameters

**Fig 7:**
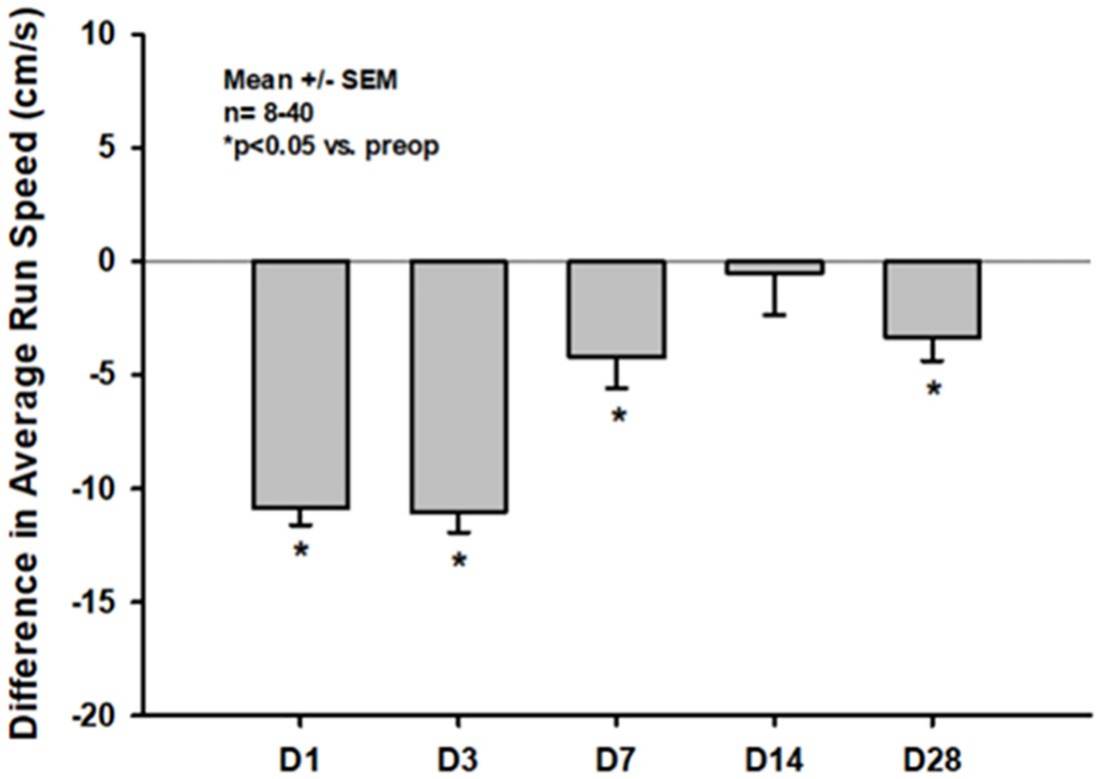
Differences in *average run speed* compared to pretrauma status on days one thru 28 after CCI.

Similar to the pattern of impairment seen in dynamic single paw parameters, *run average speed*, shown as an example for dynamic movement parameters, was significantly decreased throughout the whole observation period of four weeks (-10.87 +/- 4.85 cm/s, -11.03 +/- 5.46 cm/s and -4.22 +/- 7.02 cm/s; p<0.001 vs. pretrauma each on days one and three, p = 0.005 and p = 0.015 on days seven and 28, respectively, Fig 7).

#### Correlation between CatWalk XT^®^ parameters and lesion volume

Overall, correlation between CatWalk XT^®^ parameters and histologic lesion volume was relatively poor as average R^2^-values of all analyzed CatWalk XT^®^ parameters were only 0.20 +/- 0.02, 0.18 +/- 0.02, 0.05 +/- 0.01, 0.08 +/- 0.01 and 0.15 +/- 0.02 on days one (D1), three (D3), seven, 14 and 28 (D28) after CCI, respectively. Highest R^2^-values were observed for *max contact mean intensity* of the right hindpaw on day three (R^2^ = 0.833) and *swing speed* of the left hindpaw on day one (R^2^ = 0.831; however, only 2.0% of all R^2^-values were above 0.6 (10/500), while 90.8% (454/500) of all R^2^-values were below 0.4. Table 2 gives an overview of all R^2^-values of 0.6 or higher and the corresponding correlation coefficients.

**Table 2:** Overview of all R^2^-values above 0.6.

Parameter	Correlation Coefficient	R^2^-value
LH *swing speed* on D1	-0.91	0.831
RH m*ax contact mean intensity* on D3	-0.91	0.833
RH *mean intensity* on D3	-0.89	0.799
RH *duty cycle* on D3	-0.81	0.652
RF *max contact mean intensity* on D3	-0.81	0.650
RH *mean intensity of the 15 most intense pixels* on D3	-0.78	0.610
LF *stride length* on D28	-0.82	0.667
LH *stride length* on D28	-0.80	0.637
RF *stride length* D28	-0.79	0.627
RH *swing speed* on D28	-0.78	0.613

LH: left hindpaw, LF: left frontpaw, RH: right hindpaw, RF: right frontpaw.

**Fig 8:**
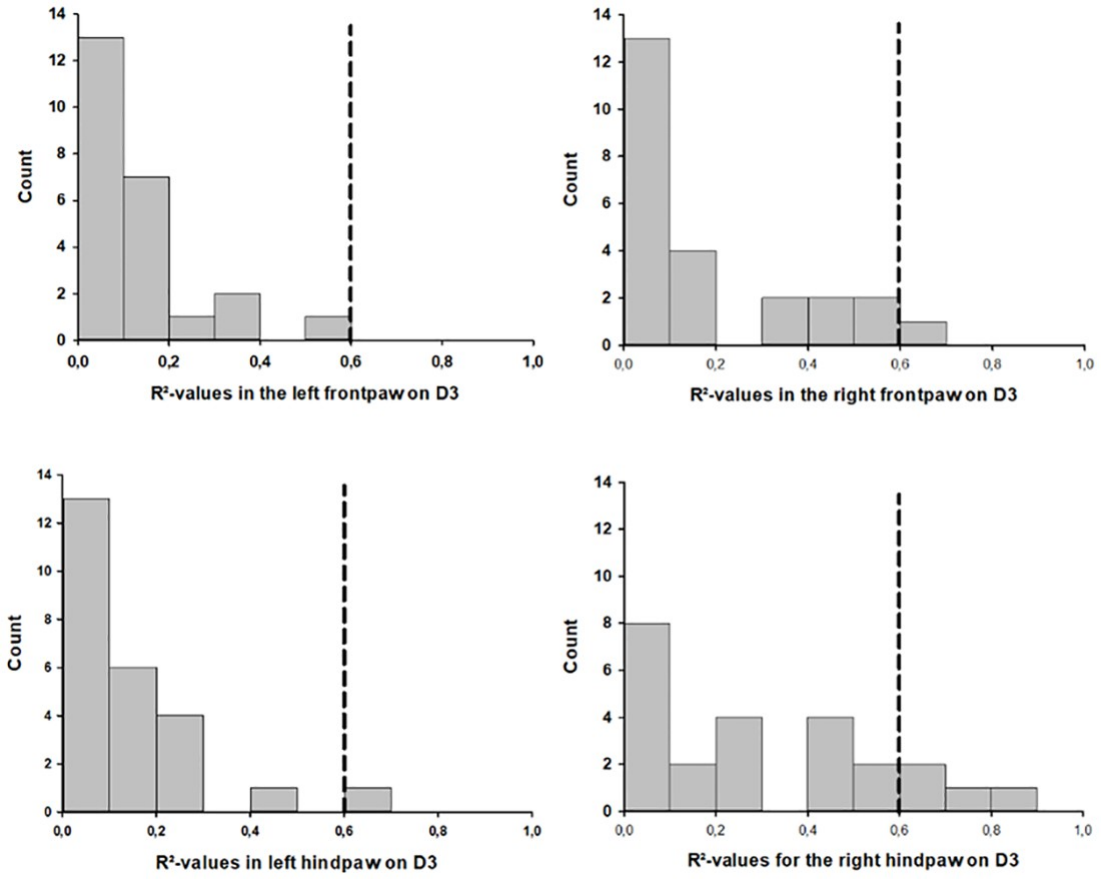
Distribution on R^2^-values for all four single paws on day three after CCI.

Even though most parameters with a correlation coefficient above 0.6 were observed on day three (5/100), there was no significant difference between timepoints concerning number of parameters with correlation coefficients of more than 0.6. There also was no significant difference in goodness of fit between left and right sided parameters, as linear regression of left sided CatWalk XT^®^ parameters on the lesion volume yielded R^2^-values above 0.6 in only 1.2% (3/250) of the cases while analysis of right sided parameters revealed R^2^-values above 0.6 in only 2.8% (7/250) of the cases. As an example, Fig 8 summarizes R^2^-value distribution of the four different single paws on day three after CCI.

## Discussion

### Temporal and spatial profile of gait impairment

In the current study we could show that CCI with the chosen parameters leads to diffuse bilateral impairments of static as well as dynamic CatWalk XT^®^ parameters in the acute phase after CCI in mice (Figs 4–7). This is well in line with multiple previous studies and might in part be explained by the fact, that many lesions extended to the corpus callosum in our current experiments [16, 34–36]. However, impairments of many static parameters including paw prints and paw intensities nearly completely resolved within the first week after trauma induction and were no longer detectable at two and four weeks after CCI (Figs 4 and 5). Previous studies in mice as well as rats have also reported a lack of gait abnormalities detectable by CatWalk XT^®^ in the chronic phase after experimental TBI; however, most of these studies reported data at multiple weeks after trauma induction only. Therefore, as gait was not evaluated in the acute phase in these studies, the exact temporal pattern of recovery of gait impairments remained unclear [37, 38]. Only one study by Henry et al., which was published at the end of 2020, systematically evaluated the temporal pattern of CatWalk XT^®^ abnormalities in the acute as well as the chronic phase after CCI in mice [39]. In this study, however, CCI did not lead to any impairment of CatWalk XT^®^ parameters at any timepoint in both, the acute as well as the chronic phase after trauma induction, at all. These results were rather surprising as it had not been reported in any previous study that CCI had not led to any gait abnormalities in the acute phase after CCI, while many studies have shown the inability of the CatWalk XT^®^ to detect gait abnormalities in the chronic phase after CCI [16, 34, 35]. Furthermore, these results are in direct contrast to the ones observed in our current study. The lack of gait abnormalities detectable by CatWalk XT^®^ in the acute phase after CCI by Henry et al. is even more surprising considering the fact that similar CCI parameters were used in the studies reporting on gait abnormalities in the first week following CCI (e.g. velocities ranged from 1.5cm/s in the study by Neumann et al. to 8m/s by Walter et al. while Henry et al. used a velocity of 6m/s). Furthermore, Henry et al. used the same piston position for trauma induction as we did in our study making a difference in trauma location unlikely to be responsible for different experimental results. As Henry et al. reported no gait abnormalities that could be detected by the CatWalk XT^®^ at any timepoint, no information on the exact timepoint of recovery of acute gait impairments could be gained from this study. Therefore, to our knowledge, our study provides information on the development of CatWalk XT^®^ detectable acute gait abnormalities within the first week after CCI and their resolution thereafter for the first time. However, we observed resolution of impairments within the first posttraumatic week in paw prints and paw intensities only, while impairment of dynamic single paw and dynamic movement parameters, such as *swing speed* and *average run speed*, persisted up to four weeks after CCI (Figs 4–7).

### Correlation between CatWalk XT^®^ parameters and lesion volume

Even though the CatWalk XT^®^ has been utilized to evaluate gait and motor function in several experimental models of TBI, data on the correlation between the impairment of CatWalk XT^®^ parameters and histological damage is still very limited and no data for the CCI model is available. The only study assessing the correlation between CatWalk XT^®^ parameters and lesion volume was published by Mountney et al. in 2013: In a model of penetrating ballistic-like brain injury in rats it was shown that at day 28 after right-sided trauma induction *swing speed* of the left forepaw, *max* and *min intensities as well as print width* of the left hindpaw and *base of support* of the hindpaws significantly correlated with lesion volume [36]. In our study, at day 28 *stride length* of both front paws as well as the left hindpaw and *swing speed* of the right hindpaw showed good correlations, defined as R^2^-values of more than 0.6, with lesion volume while none of the parameters mentioned by Mountney et al. correlated well with lesion volume. These differences might be explained by the fact, that different species as well as different models of trauma induction with different patterns of injury were used; however, in both studies, only a fraction of the analyzed parameters showed a good correlation with lesion volume on day 28 after trauma induction. In our study, this was not only true for day 28, but for all other timepoints as well. The lack of correlation between CatWalk XT^®^ parameters and the lesion volume in the acute phase after CCI indicates, that early gait disturbances after CCI cannot be thoroughly caused by the focal cortical injury alone; therefore, in addition to focal injury, they must be the result of disturbances in function of other regions of the central nervous system that are more important for gait coordination such as the cerebellum or central pattern generators located in the spinal cord. This is further supported by the fact, that lesion volume remained stable on days seven thru 28 in our study while impairments of many CatWalk XT^®^ parameters showed significant improvement with return to pretraumatic function after one week. Furthermore, CatWalk XT^®^ parameters seem to show a good correlation with lesion size in experimental models of spinal cord injury indicating the crucial role of the spinal cord in gait coordination. The lack of correlation between CatWalk XT^®^ parameters and the lesion volume in the chronic phase after CCI could, on one hand, be explained by the high plasticity of rodent motor control after focal injury, which has been shown in multiple different lesion models before: After focal lesion of the corticospinal tract, utilization of other projections such as the cortico-rubral tract seems to reorganize and restore motor function after focal unilateral injuries in rodents and cats within days while the focal injury persists; however, this compensatory plasticity seems to be less pronounced in models of TBI when compared to models of, e.g., ischemic stroke [40–44]. On the other hand, it has been shown, that a unilateral CCI leads to bilateral structural degradation, which extends well beyond the focal unilateral lesion detectable by light microscopy, and therefore, might cause functional impairments, that cannot be attributed to the focal unilateral lesion alone [45].

In our study there were no differences of the correlation between CatWalk XT^®^ parameters and the lesion volume between fore- and hindpaws or left and right paws, which at first sight might be counterintuitive considering the unilateral focal lesion induced by CCI. However, it can be explained by the fact that unilateral CCI leads to diffuse bilateral impairment of CatWalk XT^®^ parameters with no lateral accentuation in mice; therefore, one would not expect lateral differences in correlation with the lesion volume as well [16].

Our results are especially interesting because, as there is no good correlation between CatWalk XT^®^ parameters and focal lesion volume, evaluating gait using the CatWalkXT^®^ might add valuable information to only assessing histopathological damage at the injury site alone. Therefore, only assessing lesion volume at the site of CCI in preclinical studies of TBI might be insufficient to fully evaluate potential effects of a treatment as important effects might be missed.

### Timing of CatWalk XT^®^ use and choice of CatWalk XT^®^ parameters

In our study, the correlation between CatWalk XT^®^ parameters and lesion size was poor throughout all timepoints, which indicates, that assessing gait using the CatWalk XT^®^ might generate extra information on secondary brain injury after CCI throughout the first four weeks after trauma induction. However, significant impairment of all categories of CatWalk XT^®^ parameters was only seen within the first week after CCI while impairments in paw intensities and paw prints diminished thereafter. In the more chronic phase of our experiments, only dynamic parameters such as *swing speed* and *average run speed* showed persistent significant impairment. This implies, that the use of the CatWalk XT^®^ is most effective in the first week after trauma induction when using the CCI model in mice while long-term gait assessment evaluating all CatWalk XT^®^ parameters seems to be inefficient and could be limited to dynamic parameters.

### Limitations

We did not include a sham operated control group in the current study and compared intraindividual pre- and postoperative performances instead; therefore, the animals served as their own controls. This has proven to be a valid approach for automated gait analysis using the CatWalkXT^®^, as using this method, interindividual variability does not interfere with data analysis.

Even though lesion volume is one of the most utilized outcome parameters in preclinical studies of TBI and has been used in a variety of experimental TBI models, it only provides very limited information on the complex histopathological pattern of injury caused by TBI. Therefore, correlating CatWalk XT^®^ parameters to other aspects of secondary brain injury such as inflammatory processes, edema formation or induction of de- and regenerative processes would be of great interest to further evaluate the structural cause of functional impairments after experimental TBI.

Adding to this, statistical correlation of structural and functional outcome parameters cannot establish a causative relation; therefore, the data from our study is only descriptive and does not evaluate causative relations between lesion volume and gait.

Finally, the CatWalk XT^®^ only represents one of many available tests that can be used to evaluate gait and motor function in rodents. Many other functional tests such as the beamwalk, rotarod or gridwalk tests, for instance, need to be thoroughly evaluated with regards to their ability to detect even subtle impairments, their applicability in the acute as well as in the chronic phase after the initial injury as well as the correlation of functional impairments with histologic damage and therefore, the ability to provide extra information that justifies additional time and infrastructural resources that need to be utilized for extensive functional outcome assessment.

## Conclusions

In the current study we could show that gait abnormalities detected by CatWalk XT^®^ analysis do not correlate with histologically evaluated unilateral lesion volume throughout the first weeks after experimental TBI induced by CCI in mice, indicating, that gait analysis using the CatWalk XT^®^ might possibly provide valuable extra information compared to solitary histologic assessment of the site of focal injury. Our findings suggest, that preclinical outcome assessments focusing on focal histologic evaluation of the injury site alone might be insufficient as important treatment effects could be missed. Furthermore, our data revealed that the application of the CatWalk XT^®^ to assess the animals’ functional impairment after CCI is most efficient in the first week after trauma induction while gait assessment using the CatWalk XT^®^ should be limited to dynamic parameters in the chronic phase after CCI.

## Institutional review board statement

All procedures were reviewed and approved by the Animal Care Committee of the federal government (Regierungspräsidium Karlsruhe, approval number G-296/19).
